# Fragility fracture of pelvis caused by amenorrheic osteoporosis operated upon using computed-tomography-3D-fluoroscopy matching navigation system: a case report

**DOI:** 10.20407/fmj.2019-021

**Published:** 2020-03-25

**Authors:** Masahiro Yoshida, Kosuke Tajima, Koji Sato

**Affiliations:** 1 Department of Emergency Medicine, Fujita Health University Hospital, Toyoake, Aichi, Japan; 2 Department of Orthopaedic Surgery, Nagoya Daini Red Cross Hospital, Nagoya, Aichi, Japan

**Keywords:** Fragility fracture of the pelvis, Pelvic ring fracture, Amenorrheic osteoporosis, CT-3D-fluoroscopy, Intraoperative matching navigation

## Abstract

**Introduction:**

Low-energy trauma fractures of older people cause fragility fractures of the pelvis (FFPs), and secondary amenorrhea triggers osteoporosis that might lead to FFPs. Anorexia nervosa is a major causative factor in secondary amenorrhea, thus, FFPs might be a problem for young anorexia nervosa patients as well as older people. Here, we report a rare case of a young woman with anorexia nervosa who had an FFP, followed by gradual progression of severe sacral deformity.

**Case:**

A 49-year-old woman with hypothalamic amenorrhea (a subtype of secondary amenorrhea) caused by anorexia nervosa fell from a chair. She visited a nearby hospital and was diagnosed with an undisplaced sacral fracture; however, she chose to stay at home since the pain was slight and she could still walk. She fell to the floor several times while injured, and 3 months later, she had walking difficulty accompanied with severe pain, and was admitted to our facility. On radiological examination, she was diagnosed with FFP with severe sacral deformity, and was treated surgically. Because of the severe sacral deformity, a computed tomography (CT)-3D-fluoroscopy matching navigation system was used during surgery to support appropriate placement of percutaneous iliosacral (IS) and transiliac–trans-sacral (TITS) screws.

**Discussion:**

To our knowledge, this is the first report of FFP caused by amenorrheic osteoporosis, treated by matching navigation. This matching navigation could be a supportive tool in inserting IS and TITS screws during surgery for FFPs, especially in cases with severe deformity.

## Introduction

The number and age-adjusted incidence of low-energy fractures have increased considerably in the past 40 years.^1^ Low-energy trauma fractures of older people are becoming a major public health problem. Fragility fractures of the pelvis (FFPs) in older people is a recent hot topic, since inadequate treatment of FFPs may cause displacement with time or even result in nonunion.

In contrast, pelvic ring fractures in young patients occur following high-energy trauma; however, secondary amenorrhea is also known to trigger osteoporosis and might lead to FFPs. In young female athletes, especially in distance runners, there are several reports of amenorrheic osteoporosis, which has a similar high risk for FFPs.^2,3^

Anorexia nervosa is one of the major causative factors in secondary amenorrhea; thus, FFPs might be a problem for young anorexia nervosa patients as well as older people. Here, we report a rare case of a woman with anorexia nervosa who had FFP, followed by gradual progression of severe sacral deformity. The computed tomography (CT)-3D-fluoroscopy matching navigation system was used during surgery to support appropriate placement of percutaneous iliosacral (IS) and transiliac–trans-sacral (TITS) screws, with satisfactory outcome.

## Case report

**Figure 1:**
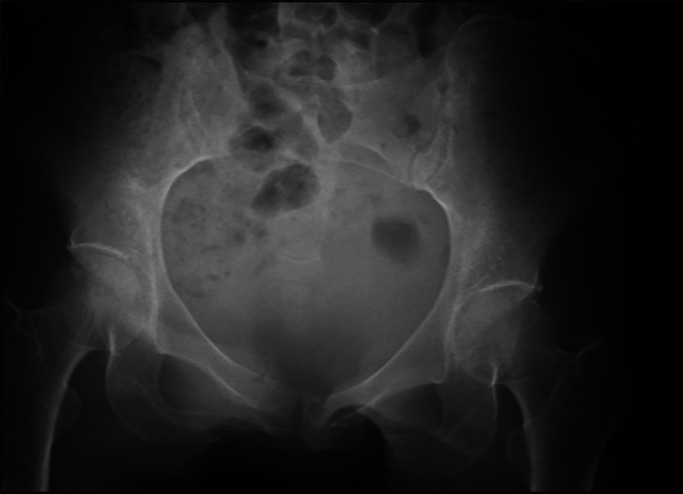
X-ray of pelvis at nearby hospital.

**Figure 2:**
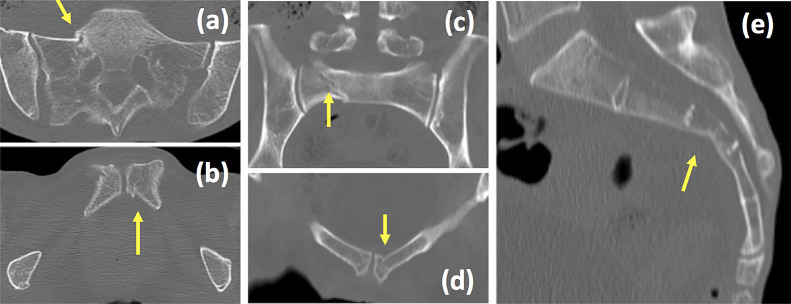
CT of pelvis at nearby hospital. Axial plane of sacrum (a) and pubis (b). Coronal plane of sacrum (c) and pubis (d). Sagittal plane of sacrum (e). Yellow arrow indicates fracture.

A 49-year-old woman with hypothalamic amenorrhea (a subtype of secondary amenorrhea) caused by anorexia nervosa fell from a chair and hit her coccyx. She visited a nearby hospital, and undisplaced bilateral rami fractures and right sacral fracture were diagnosed by X-ray investigation (Figure 1) and CT scan (Figure 2). She was diagnosed with FFP but she chose to stay at home since the pain was slight and she could still walk.

**Figure 3:**
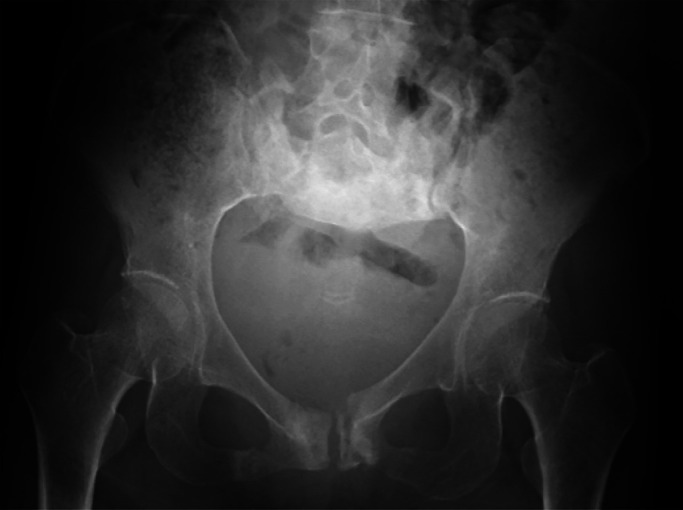
X-ray of pelvis on admission to our hospital; 3 months after the first injury.

**Figure 4:**
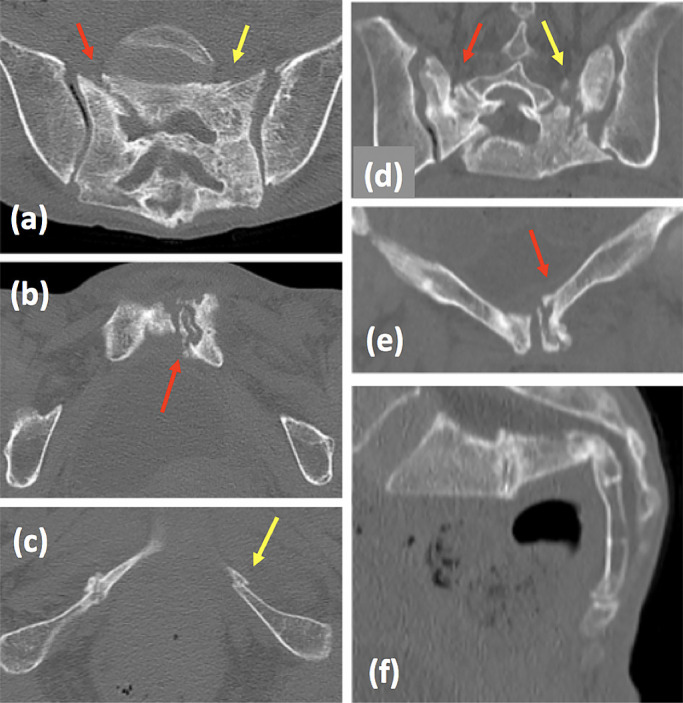
CT of pelvis on admission to our hospital. Axial plane of sacrum (a), inferior rami (b) and posterior rami (c). Coronal plane of sacrum (d) and pubis (e), and sagittal plane of sacrum (f). Red arrow indicates delayed union of former fracture; yellow arrow indicates new fracture.

**Figure 5:**
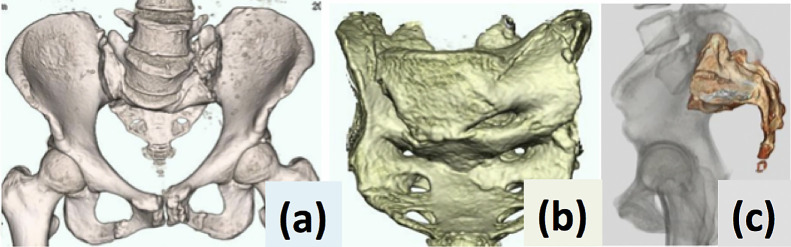
3D-CT of whole pelvis (a), anteroposterior view (b) and lateral view (c) of sacrum.

She fell to the floor several times at home after returning there. Three months later, she had walking difficulty accompanied with severe pain, and visited our facility at the Department of Emergency Medicine, Fujita Health University Hospital. X-ray examination (Figure 3) and CT scan (Figure 4) revealed delayed sacral union and a fresh fracture, accompanied with severe deformity (Figure 4f, 5), and she was diagnosed with FFP type IVb according to Rommens Classification.^4^ She was admitted to the orthopedic ward to prepare for surgery.

On admission, body mass index was 16.7 (height 149 cm and weight 37 kg), bone mineral density investigated by dual energy X-ray absorptiometry was 0.423 g/cm^2^, and Young Adult Mean (YAM) showed only 50%. On blood examination, tartrate-resistant acid phosphatase isoform 5b was 1300 mU/dL, total type I procollagen N-terminal propeptide was 399 ng/mL, and estradiol was <25.0 pg/mL.

The patient was diagnosed with FFP caused by amenorrheic osteoporosis caused by anorexia nervosa.

**Figure 6:**
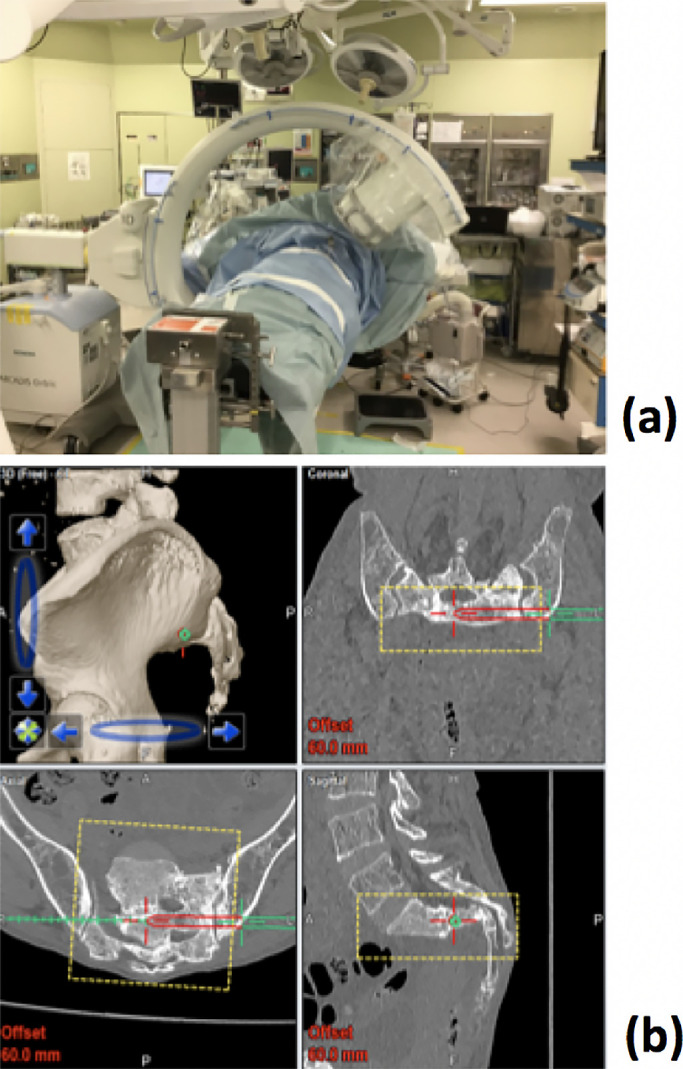
Intraoperative use of CT-3D-fluoroscopy matching navigation system (a), and screen-shot of the monitor (b).

Dietary nutrition management was immediately started, and general preoperative examination was carried out. Medication with teriparatide was also started. On planning surgery, percutaneous IS and TITS screws seemed necessary to achieve rigid stability of the left iliosacral joint. However, because of the severe sacral deformity, it was considered that conventional fluoroscopy guidance for screw placement would fail easily. Therefore, we planned to use the CT-3D-fluoroscopy matching navigation system to support appropriate placement of percutaneous IS and TITS screws (Figure 6). The navigation procedure was performed with the combination of portable 3D C-arm (ARCADIS Orbic 3D; Siemens Healthcare GmbH, Erlangen, Germany) and the navigation application (Trauma Navigation Application, Brainlab, Munich, Germany).

**Figure 7:**
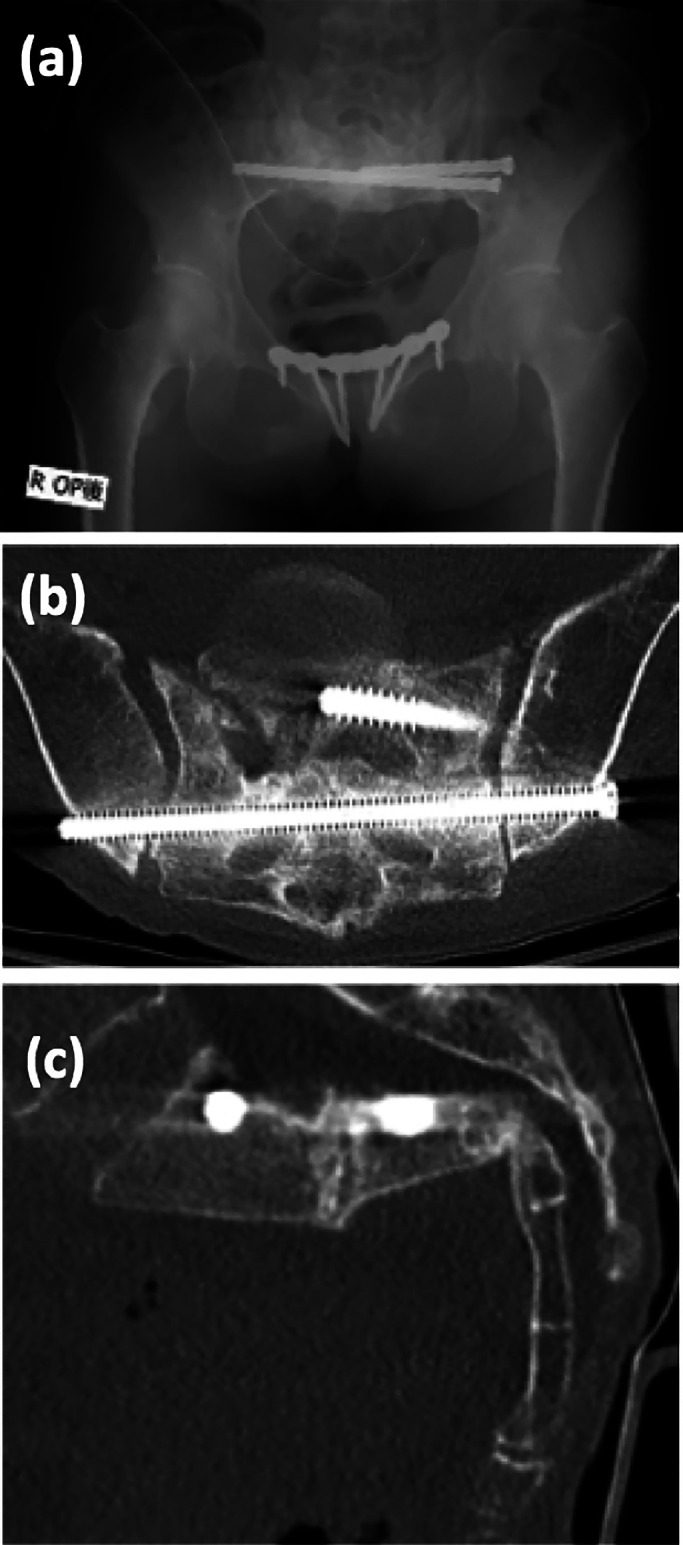
Postoperative X-ray (a), CT axial plane (b) and sagittal plane (c).

Manual examination after anesthesia revealed instability in both iliosacral joints. During surgery, preoperative and intraoperative CT images were fused, and combined with the 3D-navigation system, guide pins were placed percutaneously at the appropriate angle and depth in the supine position. Intraoperative CT imaging was performed after insertion of the guide pins to confirm the position of insertion. Finally the φ6.5 mm cannulated IS screw and TITS screw were inserted along the guide pin. After placing these screws, internal fixation of the pubis was performed using the locking plate (Figure 7).

The patient was allowed full-weight bearing from the day after surgery, and was able to walk without using a walker or crutches on day 7. She was discharged home on day 15 after surgery without complaining about any pain.

**Figure 8:**
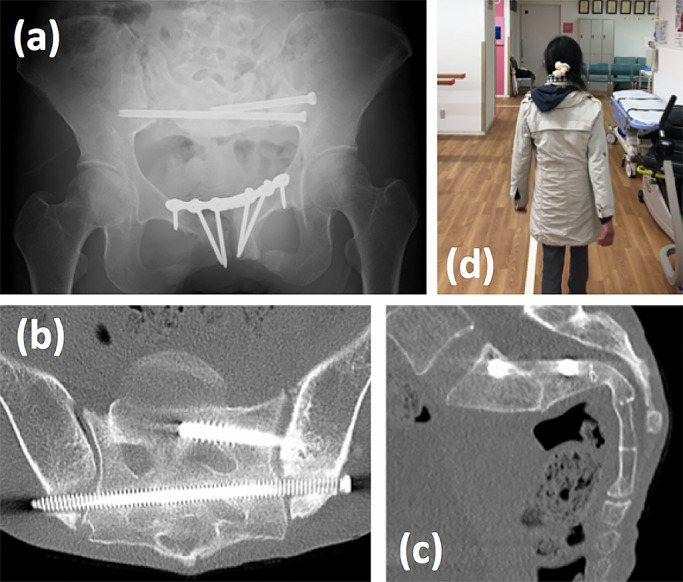
X-ray and CT 6 months after surgery. X-ray of pelvis (a), CT axial plane (b) and sagittal plane (c) of sacrum. The patient could walk without a walking aid (d).

Six months after surgery, bone union was confirmed by CT scan and she was able to walk without complaints or symptoms (Figure 8).

This report was prepared with the consent of the patient.

## Discussion

Pelvic ring fractures are usually caused by high-energy injuries such as traffic accidents and falls. However, orthopedic surgeons are currently more often confronted with FFPs; a different type of pelvic ring injury. These fractures are the result of low-energy impact or they may even occur spontaneously in patients with severe osteoporosis. In a Finnish study on individuals aged ≥80 years, the number and age-adjusted incidence of low-energy pelvic fractures increased between 1970 and 2013.^1^

Young female distance runners are similarly at high risk for pathological pelvic ring fractures, although these are not fragility fractures. These patients are subjected to cyclic vertical forces through the sacrum, resulting in pathological microarchitectural changes in bone. These bony changes are compounded by sex-hormone dysregulation, leading to FFPs.^2,3^

Furthermore, 38% of patients with anorexia nervosa are known to develop secondary osteoporosis,^5^ and 57% suffer from fractures over their whole lifetime.^6^ Thus, FFPs are usually known as low-energy trauma fractures of older people, but they may also occur in young patients with anorexia nervosa.

IS and TITS screw insertion has became a popular fixation method for unstable posterior pelvic ring injuries because the screws can be safely inserted via small surgical incisions. This minimizes the risk of operative blood loss, skin necrosis and infection associated with open procedures, and reduces operating time.^7^

Conventional fluoroscopy guidance is a standard method for percutaneous IS/TITS screw insertion. However, screw malposition rates with fluoroscopic guidance have been reported to range from 2% to 68%,^8–11^ with an incidence of neurological injury between 0.5% and 7.9%.^8,11^ To achieve appropriate screw fixation, various types of computer-assisted techniques, such as 2D fluoroscopic navigation,^12,13^ CT-based navigation,^14^ and 3D fluoroscopic navigation systems have been developed.^9,10,13^ However, 3D fluoroscopic navigation compared with conventional technique, even had a perforation rate as high as 31% in the navigated group.^9^ Appropriate placement of IS and TITS screws without perforation of the sacrum or sacral foramen is difficult, even with the use of 3D fluoroscopic navigation. The CT-3D-fluoroscopy matching navigation system could reduce the malposition rate of percutaneous IS/TITS screw insertion, even when performed by less-experienced surgeons.

Our case was a young woman with anorexia nervosa who had FFP, followed by gradual progression of severe sacral deformity. The CT-3D-fluoroscopy matching navigation system was used during surgery to support appropriate placement of percutaneous IS and TITS screws, and satisfactory outcomes were obtained. Although the patient is currently able to walk without complaints or symptoms, it is controversial whether or not the remaining sagittal malalignment will cause any problem during long-term follow-up.

To our knowledge, this is the first report of FFPs caused by amenorrheic osteoporosis caused by anorexia nervosa, treated surgically with matching navigation. Although there are several reports indicating the utility of matching navigation during treatment of FFPs,^15,16^ none of them mentions the merit of matching navigation in cases of severe deformity. The matching navigation system described here could be a supportive tool for insertion of IS and TITS screws during surgery for FFPs, especially in cases with severe deformity.
